# When does binding become learning, if it ever does? How sequences of stimulus–response combinations affect episodic retrieval in a color-word repetition paradigm

**DOI:** 10.3758/s13423-025-02837-9

**Published:** 2026-01-08

**Authors:** Matthäus Rudolph, Klaus Rothermund

**Affiliations:** Department of Psychology, Friedrich Schiller University Jena, General Psychology II, Am Steiger 3, Haus 1, D-07743 Jena, Germany

**Keywords:** Episodic binding and retrieval, Event coding, Learning, Memory

## Abstract

Current theories about binding and learning claim that transient episodic bindings between stimulus and response features serve as the foundation for forming long-term stimulus–response (S-R) associations in memory. In two high-powered, pre-registered experiments (total *N* = 163), we observed that the stimulus–response binding and retrieval effect increased linearly with each additional episode contributing to the uninterrupted repetition of the same S-R combination. To examine whether this repeated exposure results in the formation of an abstract, stable, nonepisodic S-R association in long-term memory, we tested whether the influence of the uninterrupted series persists after a single intervening episode that contradicts the series. Our results show that the repetition effect does not modulate retrieval effects for S-R combinations that deviate from the series and thus does not survive a single mismatching episode, even after a large number (i.e., 10 or 11) of prior repetitions. Hence, the increased retrieval effect for long series of matching episodes does not reflect a transition from episodic retrieval to long-term learning but may instead reflect a higher probability of successfully retrieving a matching S-R episode from memory. In sum, we found no convincing evidence that pure S-R repetitions in and of themselves (independently of other processes like hypothesis testing or propositional reasoning) lead to the formation of a stable, abstract, nonepisodic representation (i.e., an association) that operates independently of binding and retrieval.

Feature binding and retrieval are central mechanisms in human action control. During *binding*, features are integrated into an event file, which is a hypothetical episodic memory structure that integrates a wide range of information, such as stimulus features (e.g., color, position, shape, size, orientation), response features (e.g., duration, location, body part), and anticipated consequences of a response (Hommel, 1998, 2004). Subsequent repetition of one of these features typically leads to an automatic *retrieval* of the other features of the event file, which then can have an impact on performance in the new situation. Specifically, whenever a response is executed to a stimulus, the mental codes of the stimulus and the response become integrated, resulting in episodic stimulus–response (S-R) bindings (synonymous terms include S-R episode; Frings et al., 2024) that are stored in memory. As soon as the stimulus is encountered again, the response is retrieved automatically, which facilitates responding if the same response is required, but impedes responding if a different response is required (e.g., Frings et al., 2007; Hommel, 1998; Rothermund et al., 2005).

Learning can be defined as a relatively stable change in behavior that is rooted in regularities (e.g., between stimuli and responses) and facilitates adequate and efficient responding to situations that are consistent with these learned regularities, compared with those that are not (De Houwer & Hughes, 2020). In the present paper, we provide an experimental approach to investigate a potential relationship between binding and learning.

Current theories about the relationship between learning and binding claim that transient episodic stimulus–response bindings could be the building blocks for long-term S-R associations in memory (Frings et al., 2023): In accordance with the idea that behavior automatization develops under conditions of a “consistent mapping” of stimuli and responses (Schneider & Shiffrin, 1977), and findings showing that the sheer number of repetitions of a specific S-R combination establishes a connection that renders the behavior habitual, temporally stable, and independent of expected rewards (Adams, 1982; Dickinson, 1985; for more recent evidence, see Schmidt, De Houwer, et al., 2020a, 2020b), the authors speculated that the strength of the retrieval effect increases with the number of successive, uninterrupted repetitions of a stimulus (S) in combination with the same response (R). Through the continuous, closely timed repetition of S-R pairings, the binding strength between stimulus and response should become amplified, until a critical threshold may be reached, facilitating the transition of episodic S-R bindings into long-term S-R associations within semantic memory (Logan, 1988; Logan & Etherton, 1994). If, however, the S-R pairing is not reencountered within a short timeframe, the S-R binding is likely to decay completely, thereby preventing its integration into long-term memory (Frings et al., 2023). According to this account, the establishment of lasting mental representations that are the source of long-term learning builds upon what was initially a *retrieval* effect.

One should also consider the possibility, however, that the assumption of a relation between successive repetitions of a certain S-R combination and establishing enduring memory traces that underlie long-term learning might actually not hold true, since (a) there is no direct evidence supporting this claim yet, and (b) several studies suggest that binding and learning can be considered as two distinct processes (Colzato et al., 2006; Dames et al., 2022; Moeller & Frings, 2017; Rothermund et al., 2025). In accordance with this view, the strength of episodic binding and retrieval effects has been shown to be unaffected by various factors that are known to influence learning. For instance, the strength of episodic S-R binding and retrieval effects is not modulated by affective consequences (Martini et al., 2025; Mocke et al., 2025; Parmar & Rothermund, 2023; Schöpper et al., 2025), nor is it influenced by contingency awareness (Rudolph et al., 2025), although both factors play an important role in shaping operant or Pavlovian conditioning (e.g., Lovibond & Shanks, 2002; Mitchell et al., 2009; Rescorla, 1972; Rozin & Fallon, 1987; Schiller et al., 2008; Thorndike, 1911).

To our knowledge, no previous study directly tested the influence of successive repetitions of an S-R combination on the strength of binding and retrieval effects, nor does any evidence exist regarding the emergence of a stable “associative” representation of the S-R combination in long-term memory after repeated encounters of the same S-R combination. Providing such evidence is relevant for evaluating the theoretical claim that repeated encounters of the same S-R combination will eventually result in a lasting associative S-R link that is the source of long-term learning (Frings et al., 2023).

To provide an empirical test for this theoretical claim, we introduce a novel version of the color-word categorization task that was specifically designed to systematically manipulate the number of successive S-R repetitions (i.e., the number of repetitions of specific color-word pairings). This novel paradigm allows a test whether the number of successive repetitions of a specific S-R combination modulates the strength of the resulting binding and retrieval effect. Furthermore, to test whether the length of the uninterrupted series of repeated encounters with a specific S-R combination establishes a permanent “associative” S-R link in long-term memory, the paradigm also allows us to test whether an effect of the number of repetitions survives an intervening episode that deviates from the series. If episodic S-R bindings truly function as the foundational building blocks for the formation of long-term S-R associations in memory, the accumulation of S-R episodes should (a) increase the strength of the binding and retrieval effect for the last episode of the series, and should (b) exert a lasting effect on behavior, even after encountering an intervening episode in which the stimulus is paired with a different response.

Note that, in contrast to the typical color-word contingency learning paradigm (Schmidt et al., 2007) in which words appear with a high frequency in a particular color and less often in other colors, the color-word repetition paradigm introduced in this study includes no global contingencies between colors and words. The focus is on testing whether a series of uninterrupted repetitions of the same S-R combination, in and of its own, leads to the establishment of a lasting, associative representation in long-term memory. Implementing contingencies introduces a confound with repetitions, since a contingency implies that specific S-R combinations are more (or less) likely to (re-)occur. Previous studies have shown that introducing experiment-wide overall contingencies invites additional processes of propositional reasoning and hypothesis testing that are drivers of learning and performance (e.g., contingency awareness and contingency beliefs; Giesen et al., 2025; Rothermund et al., 2025; Rudolph et al., 2025; Rudolph & Rothermund, 2025). To investigate pure effects of repetitions, independent of propositional reasoning and hypothesis testing, we avoided any confounds between repetitions of specific S-R combinations and overall contingencies, and we chose to examine the pure effects of S-R repetitions over relatively short time spans.

## The present study

**Fig. 1:**
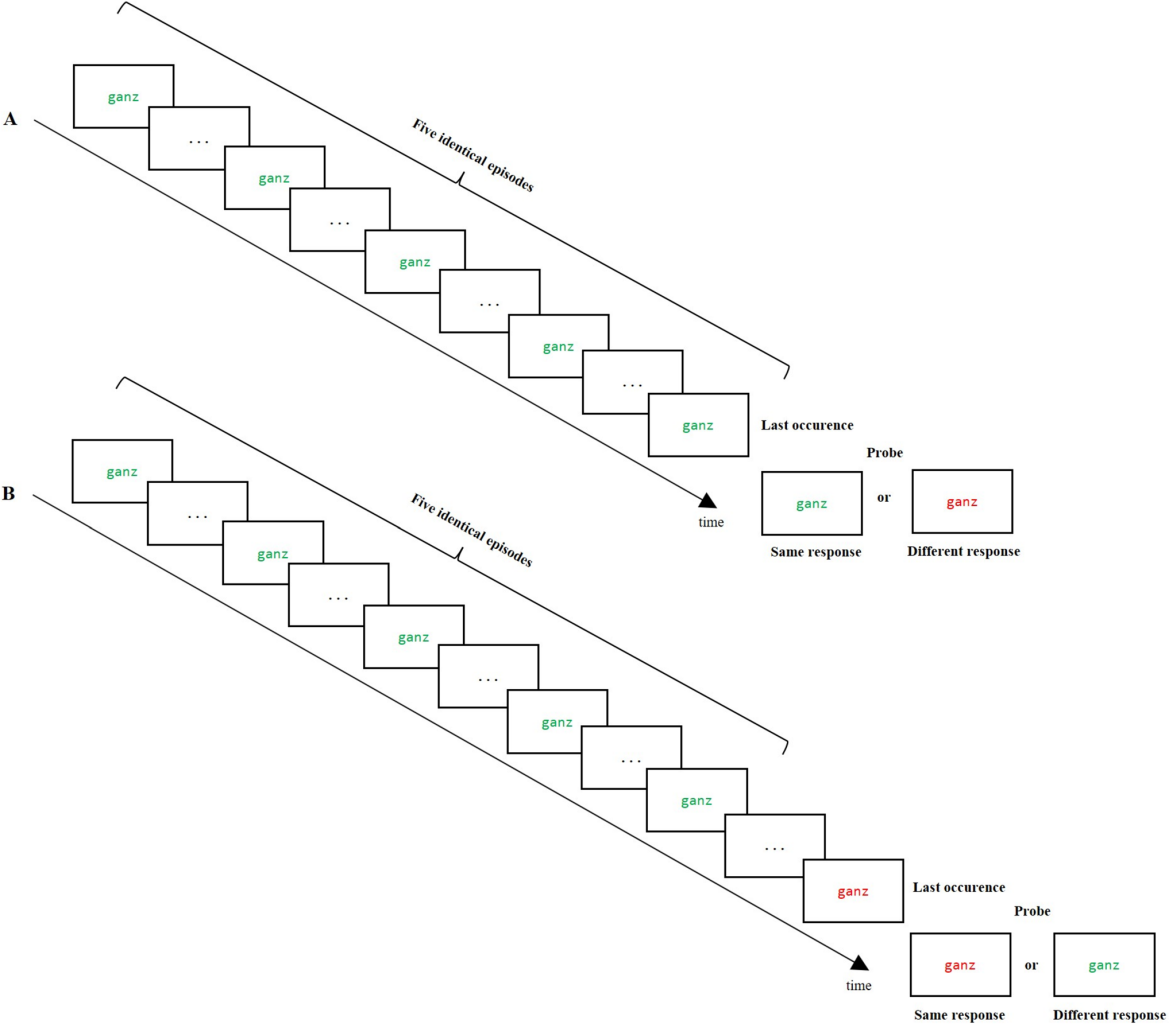
Exemplary trial sequence in the color-word repetition task. By stimulus-based retrieval, the response which was bound to the word stimulus during its *last occurrence* becomes reactivated which facilitates responding in the probe if the same response is required and interferes with responding if a different response is required. **A** *Modulation of response retrieval effects by the number of prior repetitions.* A higher number of prior repetitions of both word and color response should amplify the response retrieval effect (Hypothesis 2). **B** *Modulation of response retrieval effects for a mismatching episode.* In this example, five identical episodes in which the word was paired with a different response preceded the last occurrence of a word stimulus. If the uninterrupted repetition of an S-R episode has a lasting effect on responding, the retrieval effect of a single episode (i.e., the last occurrence effect) should be weakened by the series of deviating S-R episodes that immediately preceded the last occurrence (Hypothesis 3)

The main goal of the present study is to investigate the modulation of the strength and stability of retrieval effects by systematically manipulating the number of successive repetitions of stimulus and response features^1^ preceding a retrieval cue. Specifically, we test the following hypotheses:

1. We expect a response retrieval effect, characterized by faster responding if the most recent occurrence of the same stimulus required the same response, compared with trials where the most recent occurrence of the stimulus required a different response (“law of recency”; Giesen et al., 2020; Schmidt, Giesen, et al., 2020a, 2020b).
2. We investigate whether the magnitude of the response retrieval effect is influenced by the number of previous successive identical repetitions that immediately precede the last occurrence (i.e., the number of prior repetitions of the word in the same color). If the number of prior repetitions modulates this effect, an increased number of prior repetitions of both stimulus and response should amplify the response retrieval effect—that is, the difference between response repetition and response change trials (see Fig. 1A).
3. To test whether the number of uninterrupted repetitions of a specific S-R combination has a lasting effect, resulting in an abstract, nonepisodic representation of this combination, we investigate whether the number of repetitions still influences binding and retrieval after one intervening episode in which the stimulus is paired with a different response. Specifically, we test whether the retrieval effect of a single episode (i.e., the last occurrence effect) is weakened by the length of a homogeneous series of mismatching S-R episodes that immediately precedes the last occurrence. If repetitions of the same S-R episode have a lasting effect that survives an intervening episode, then the retrieval effect of the last episode should become weaker with additional elements of a preceding series of repeated mismatching episodes (see Fig. 1B).

## Experiment 1

### Method

#### Ethics vote, preregistration, and open access

Ethical approval was granted by the universities Ethics Committee. Prior to data collection, the method, design, hypotheses, data preparation, and planned analyses were pre-registered online (https://aspredicted.org/3kcs-zqpf.pdf). The experiment, data, and analysis script are available online (https://osf.io/p45ye/?view_only=86367fc25b0c48f5bbb98b7baa8a9319).

#### Required sample size and a priori power calculation

An a priori power analysis using G*Power (Version 3.1.9.6; Faul et al., 2007) revealed that at least *N* = 71 participants are necessary to identify a small to medium effect (*d*_z_ = .3) corresponding to a linear modulation of the retrieval effect by the number of successive repetitions in a one-tailed test with a minimum power of *ß* = .80. For Experiment 2, the same power analysis parameters were used to determine the required sample size, ensuring comparable sensitivity across experiments.

#### Participants

A total of *N* = 92 psychology students (10 men, 80 women, two diverse) with a mean age of 20.82 years (*SD* = 2.42) signed up and took part in the experiment, which was conducted online via E-Prime Go. Participants were recruited online and received a link via a mailing list, which allowed them to participate in the study. All participants had a mean error rate below 20% in the practice block and within the experimental blocks. Thus, the data of all participants could be processed. All participants were native German speakers. They gave their informed consent prior to participation in the study and received a partial course credit for participation. The experiment lasted approximately 30 min.

#### Design

**Fig. 2:**
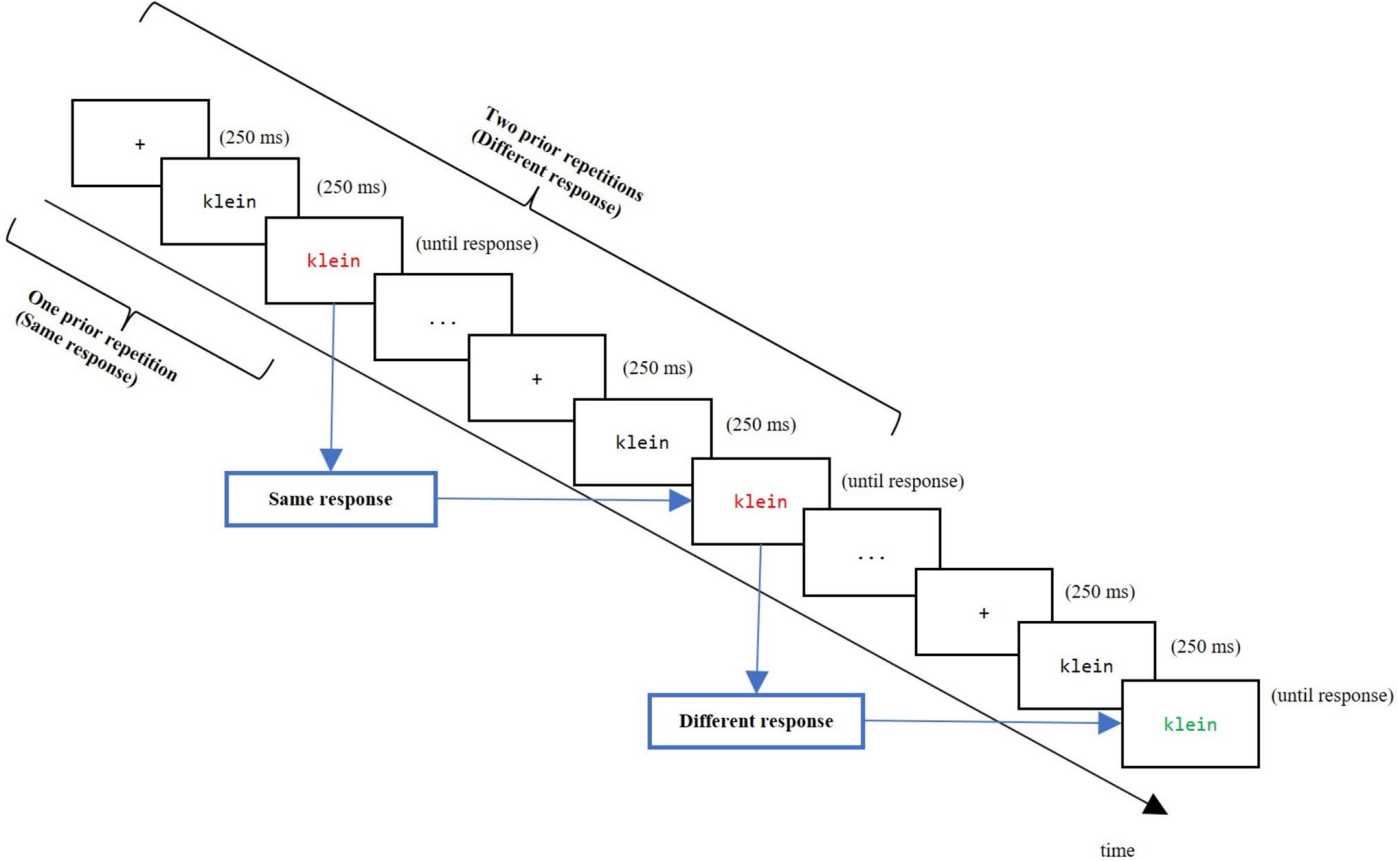
Experimental design. Arrows illustrate different types of response relations between the current trial and the last occurrence of the same distractor word (same response, different response). The number of prior repetitions indicates how often a word appeared in the same color before the retrieval event. Note that only trials containing one of the two possible words are shown to illustrate the factorial design. In the original study, white/colored words were presented on black background

The experiment comprised a 2 (response relation to the last occurrence of the same word: same vs. different response) × 5 (number of prior repetitions: 1 vs. 2 vs. 3 vs. 4 vs. 5) repeated-measures design, with all factors being manipulated within participants (see Fig. 2 for an illustration of the response relation factor and the prior repetition factor). Reaction times (RT) functioned as the dependent variable of interest.

To manipulate response relation, each trial is categorized based on whether the same or a different response was required during the last occurrence of the distractor word (factor “response relation”; see Fig. 2). Additionally, each retrieval event is classified according to the number of successive identical repetitions that immediately preceded the last occurrence, defined as the number of prior repetitions of the word in the same color.

#### Apparatus, stimuli, and sequencing

**Fig. 3:**
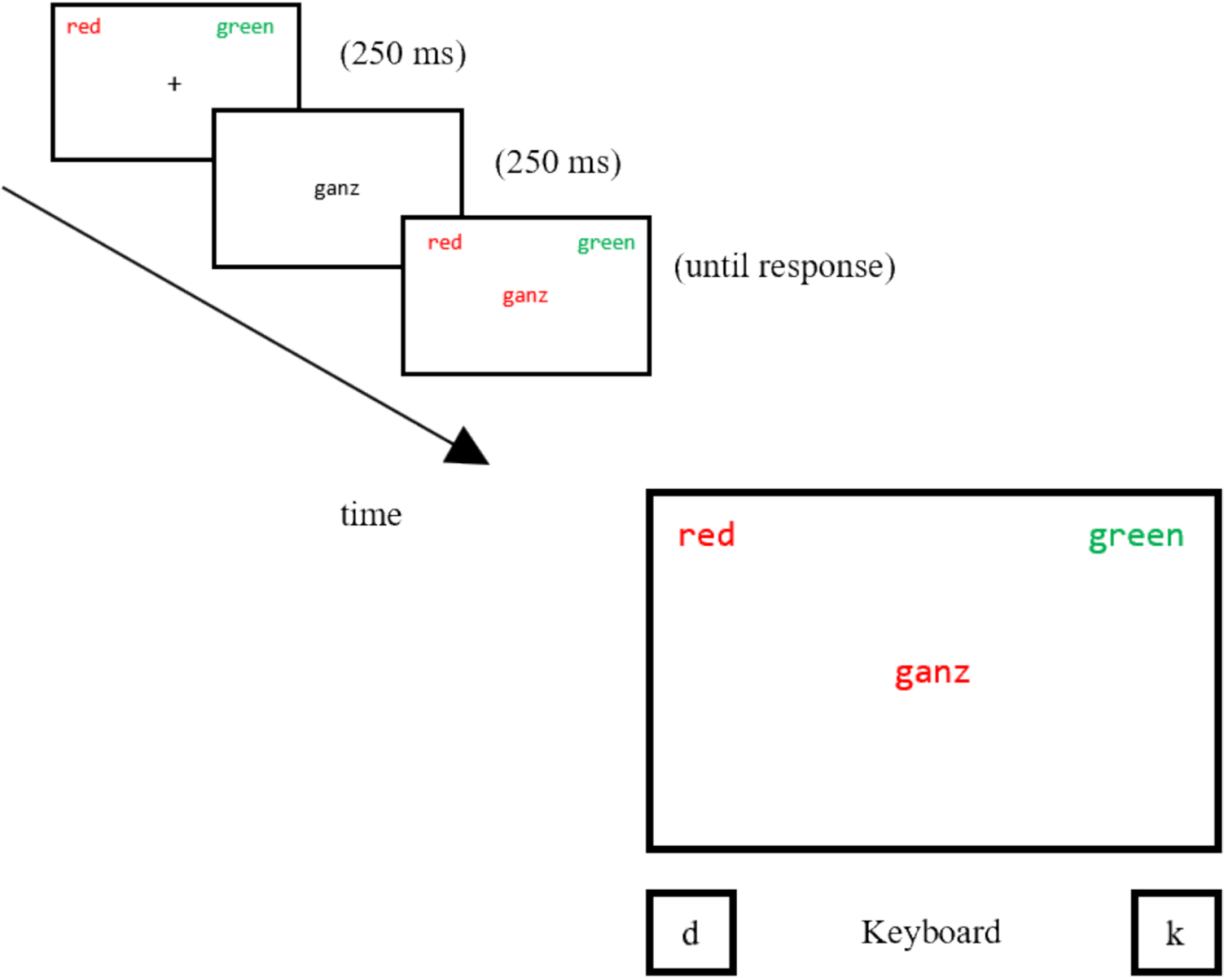
Exemplary trial sequence in the color classification task. In the original study, the color labels were printed in German and white/colored words were presented on black background

The Experiment was programmed with E-Prime 3.0. Stimuli were two neutral German adjectives (klein [small], ganz [whole]) which appeared in one of two colors (red, green), resulting in 2 × 2 = 4 color-word combinations. Stimuli were presented in Consolas font (18 pts.) on a black background. The keyboard served to collect responses. Each of the two target colors was assigned to one response key. Specifically, participants should use the index fingers of both hands to press the “d” (left) and “k” (right) keys to classify the colors of the words (see Fig. 3).

To generate the final list of words in specific colors for each participant, *two separate lists were created*—one for each of the two words used in the study. Each word-specific list contains 160 sequences of length one, 80 sequences of length two, 40 sequences of length three, 20 sequences of length four, 10 sequences of length five, and 10 sequences of length six. Half of these sequences are red, and the other half are green (counterbalanced with length of the sequences). A sequence refers to consecutive presentations of the same word in the same color. From these existing sequences, we drew sequences in alternating colors without replacement, starting with either a red or a green sequence at random. Because there is an equal number of sequences for each color, this alternating procedure continues until all sequences are used, resulting in two perfectly balanced word-specific lists.

From the two ordered, word-based lists (Word 1 list and Word 2 list), each comprising 630 trials, we constructed a final list of 1,260 experimental trials. The final list was generated by sequentially progressing through both source lists from beginning to end, and drawing trials from each of the two lists in a randomly alternating fashion. Thus, each of the 1,260 positions of the final list was determined by randomly drawing the next trial from either the Word 1 or the Word 2 list (with a 50% probability for each of the two lists). By working through the two word lists from beginning to end, this procedure preserved the lengths of the sequences of identical color-word combinations from each of the two source lists. In the final list, sequences of identical color-word combinations for Word 1 and Word 2 were interleaved. Accordingly, in the final list, sequence length is determined by counting the number of trials in which the current word had consecutively appeared in the same color without being presented in a different color, ignoring trials during which the other word had been presented. This sequencing procedure, with the numbers of sequences of each length specified above, ensured that (a) each color-word combination occurred with equal overall frequency, and (b) the total number of response repetitions (same response trials) and response alternations (different response trials) was identical for each sequence length.^2^ Lists were generated individually so that each participant received a unique set of trials.

#### Procedure

Participants first read a primer of the study content and requirements. Demographic information (age, gender, handedness) was gathered at the beginning of each experiment, followed by the consent page. If participants consented to participate, instructions were given; otherwise, the study was terminated. Participants were informed that they will see words which appear in different colors and that their task is to categorize the color of the words. Participants were asked to keep the index fingers of both hands above the response keys and to react as fast as possible. Further, they were informed that the experiment consists of one practice block and six experimental blocks.

After reading the instructions, participants worked through the practice block that was identical to the experimental blocks and consisted of 10 trials. Participants had to repeat the practice block if they committed more than 20% errors. If the error rates still exceeded 20% after the repetition, the experiment was aborted. After completing the practice block, participants worked through six experimental blocks, whereby each block consisted of 210 color-word combinations (1,260 trials in total). After finishing the experiment, participants were given a code that served as a proof for successful completion. When they entered the code in the online portal, participants were rewarded accordingly.

Every trial started with a fixation cross (250 ms) followed by a short presentation of the distractor word in white (250 ms), after which the final color-word combination appeared until key press. During both the practice and experimental blocks, incorrect responses elicited the feedback “Error—be more accurate! Continue with space-bar. ..”. Feedback was displayed in white font on red background until key press. Afterwards, the next trial started immediately. Throughout the experiment, the assignment of colors to corresponding responses (left or right) was displayed on the upper left and upper right side of the screen (see Fig. 3).

#### Data preparation

Prior to analysis, the first trials of each block (0.5%) and trials with erroneous responses (3.2%) were excluded. Next, we excluded all trials that were preceded by trials with erroneous responses or with one (or more) error(s) occurring during one of the preceding repetitions of the respective word (2.7%). Finally, we eliminated outliers by removing all trials with RTs that were below 150 ms or that were more than 3 interquartile ranges above the third quartile of the individual RT distribution (1.7%; “far out values” according to Tukey, 1977). The practice block was omitted from the analysis.

### Results

We conducted a hierarchical multi-level analyses based on individual trials, treating trials as nested within subjects, while allowing for random intercepts to control for differences in mean RTs between participants. RT served as the dependent variable of interest. Predictors were successively added to the models. All predictors indicating a contrast between conditions (i.e., response retrieval [RR], number of prior repetitions of the word in the same color [NR]) were coded to have a mean of zero across all trials and a difference of 1 between the weights. Thus, the resulting regression coefficients reflect the difference in average response times between the two conditions in milliseconds.^3^

To assess response retrieval effects, we focus on the contrast between trials in which the probe word was presented in the color that shared the *same response key* for this word during its last occurrence (same response, coded as +.51), and trials in which the probe word was presented in a color that was assigned to a *different response key* during its last occurrence (different response, coded as −.49). To model both linear and potential non-linear changes in response retrieval effects as a function of the number of prior repetitions of color and word, we constructed linear, quadratic, cubic, and quartic polynomial contrasts based on the NR factor.

#### Response retrieval effects

In the first step, we tested the effect of episodic retrieval of previous response information from the most recent occurrence of the same stimulus. Including response retrieval as the only level 1 predictor yields a large and highly significant retrieval effect, *β* = −28.34, *t*(104,122) = −41.01, *p* < .001. On average, participants responded 28-ms faster if the most recent occurrence of the word required the same response, compared with trials where the most recent occurrence of the word required a different response.

#### Modulation of response retrieval effects by the number of identical repetitions

**Fig. 4:**
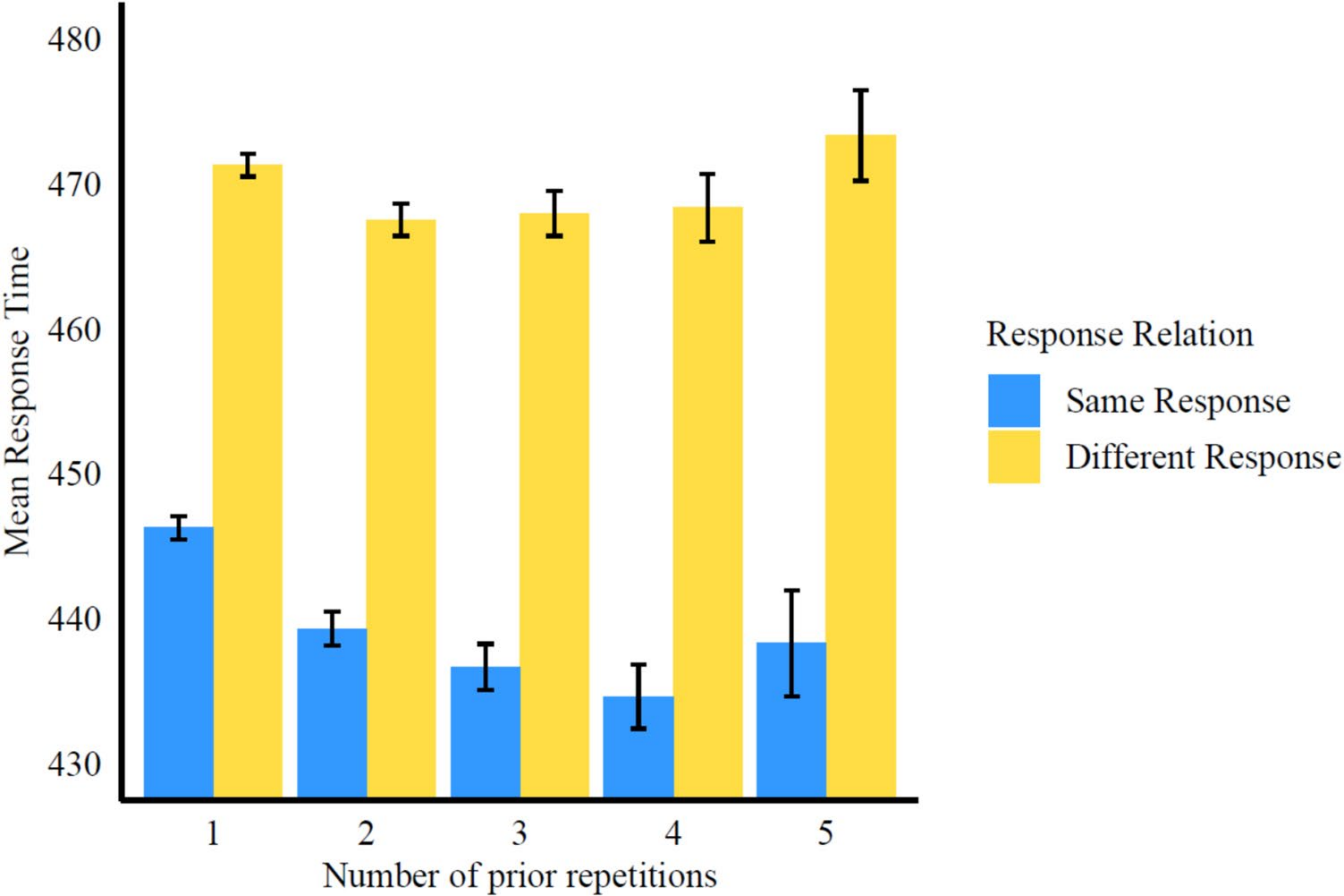
Mean response times as a function of response relation (same vs. different response) and number of identical prior repetitions of the S-R episode (1 vs. 2 vs. 3 vs. 4 vs. 5). The bars represent standard errors. The response retrieval effect is reflected in the difference in height between the bars representing same and different response trials

To test whether the response retrieval effect is modulated by the number of previous repetitions of color and word, we added the linear, quadratic, cubic, and quartic polynomial contrasts and their interaction with response retrieval to the model. We obtained a significant product term for the response retrieval and linear polynomial interaction, *β* = −3.16, *t*(104,120) = −4.88, *p* < .001 (see Table 1 and Fig. 4 for the descriptive results). This indicates that on average, the size of the response retrieval effect increases by an estimated 3 ms with each additional prior repetition of the word in the same color.^4^ All other interactions of higher order polynomial contrasts and the retrieval effect were not significant (all* t* ≤ 0.50 and ≥ −0.30, *p* ≥ 0.616).

**Table 1:** Mean reaction times and standard deviations in milliseconds depending on episodic response retrieval (retrieval of a same vs. different response) and the number of uninterrupted prior repetitions of the word in the same color (1 vs. 2 vs. 3 vs. 4 vs. 5)

	Number of uninterrupted prior repetitions				
	1	2	3	4	5
SR	446 (133)	439 (132)	437 (128)	434 (123)	438 (141)
DR	471 (133)	468 (132)	468 (128)	468 (135)	473 (128)
RR	−25 (188)	−29 (186)	−31 (181)	−34 (183)	−35 (190)

SR = same response. DR = different response. Standard deviations are reported in parenthesis. RR effect: RT_SR_ – RT_DR_

#### Influence of the number of repetitions on retrieval effects for a deviating episode

To examine whether repeating the same S-R combination eventually leads to a stable representation that influences responding independently of the last occurrence, we conducted a second analysis. In this analysis, we tested whether the response retrieval effect of a single episode is weakened by a series of preceding S-R episodes in which the word stimulus was paired with a *different color* (see Fig. 1B). For this analysis, we included only probe trials in which the color-word combination presented during the last occurrence of the word repeated only once (52.4% of all trials). To model both linear and potential non-linear changes in response retrieval effects as a function of the number of mismatching S-R episodes that immediately preceded the last occurrence, we constructed linear, quadratic, cubic, and quartic polynomial contrasts based on the number of mismatching episodes prior to the last occurrence of the word.

Including response retrieval as the only level 1 predictor results in a large and highly significant retrieval effect, *β* = −25.64, *t*(53,497) = −26.3, *p* < .001, indicating that participants responded 26-ms faster if the most recent occurrence of the word required the same response, compared with trials where the most recent occurrence of the word required a different response. To test whether the response retrieval effect is modulated by the number of mismatching episodes preceding the last occurrence of the word, we included linear, quadratic, cubic, and quartic polynomial contrasts and their interactions with response retrieval in the model. The interaction terms yielded no significant effects (all* t* ≤ 0.55 and ≥ −0.99, *p* ≥ .318), indicating that the response retrieval effect remains robust to a preceding series of repeated presentations during which the word stimulus was presented in a different color. In particular, there was no modulation of the strength of the retrieval effect by the linear contrast coding the number of repetitions in a different color that preceded the last occurrence of the word, *β* = −0.56, *t*(53,496) = −0.73, *p* = .465. To further assess the evidence for the absence of this interaction, we computed a Bayes Factor (*BF*) comparing the model with the two-way interaction to the model without it. The *BF₀₁* indicated moderate evidence in favor of the simpler model without the two-way interaction (*BF₀₁* > 9.63), suggesting that the interaction is not needed to account for the data. Hence, the number of uninterrupted repetitions of a specific S-R combination has no lasting impact on responding, as it fails to persist through even a *single* intervening episode that is contrary to the series.

### Discussion

In Experiment 1, we observed larger response retrieval effects for longer compared with shorter color-word sequences. This repetition effect disappeared as soon as a single color-word combination contrary to the sequence was presented, making the formation of an abstract association unlikely. However, it remains possible that five consecutive presentations of a word in the same color are insufficient to establish a long-lasting, nonepisodic representation. Therefore, we conducted a second experiment to provide a more stringent test of our hypotheses by doubling the length of the sequences.

## Experiment 2

### Method

#### Ethics vote, preregistration, and open access

The method, design, hypotheses, data preparation, and planned analyses of Experiment 2 were preregistered online prior to data collection (https://aspredicted.org/n27x-2kpv.pdf). The experiment, data, and analysis script are available on OSF (link provided as in Experiment 1).

#### Participants

A total of *N* = 71 participants (18 men, 50 women, three diverse) with a mean age of 22.9 years (*SD* = 3.91) signed up and took part in the experiment, which was conducted online via E-Prime Go. Most participants (*N* = 49) were students recruited online via a mailing list and received partial course credit as compensation. The remaining participants (*N* = 22) were recruited via Prolific and received £5.50 for their participation. Note that both groups showed a similar pattern of results (see Footnote 2). All participants had a mean error rate below 20% in the practice block and within the experimental blocks. Thus, the data of all participants could be processed. All participants were native German speakers who gave their informed consent prior to participation in the study. The experiment lasted approximately 45 min.

#### Design

The experiment comprised a 2 (response relation to the last occurrence of the same word: same vs. different response) × 5 (number of prior repetitions: 1 vs. 10) repeated-measures design, with all factors being manipulated within participants. Reaction times (RTs) served as the dependent variable of interest.

#### Apparatus, stimuli, and sequencing

The methods of Experiment 2 were identical to those of Experiment 1, except for the following changes. In Experiment 2, most trials had a sequence length of one, 10, or 11. To make the occurrence of specific word-color combinations less predictable, lists with intermediate length (i.e., three to nine) were randomly interspersed into the sequence, but with lower frequency. Specifically, each word-specific list contained 40 sequences of length one, 40 sequences of length ten, and 40 sequences of length eleven. In addition, two sequences were included for each sequence length between two and nine (i.e., two sequences of length two, two sequences of length three, […], two sequences of length nine), resulting in a total of 968 trials per list.

For Experiment 2, the final list of 1,936 trials was constructed similarly to Experiment 1: trials were drawn in randomly alternating fashion with a 50% probability from the two ordered word-specific lists, starting at the beginning and then proceeding until the end of both lists, until all trials from both source lists were included. This procedure ensured that each color-word combination occurred with equal overall frequency while allowing systematic manipulation of sequence length. Note that, in this case, the final participant-specific list contained overall more response repetitions (same response trials) than response alternations (different response trials), because implementing longer sequences with higher frequency led to more color-word combinations that required the same response as the last occurrence of the word.

#### Procedure

The general procedure (instructions, practice block, color-categorization task, and trial structure) was identical to that in Experiment 1. However, Experiment 2 was significantly longer, comprising eight experimental blocks of 242 color-word combinations each. Upon finishing the experiment, participants were given a code as proof of successful completion. When they entered the code in the respective online portal, participants were rewarded with course credit or monetary compensation, accordingly.

#### Data preparation

Prior to analysis, the first trials of each block (0.4%) and trials with erroneous responses (3.2%) were excluded. Next, we excluded all trials that were preceded by trials with erroneous responses or with one (or more) error(s) occurring during one of the preceding repetitions of the respective word (14.3%). Finally, we eliminated outliers by removing all trials with RTs that were below 150 ms or that were more than 3 interquartile ranges above the third quartile of the individual RT distribution (2% “far out values”; Tukey, 1977). The practice block was omitted from the analysis.

### Results

To analyze the data in Experiment 2, we conducted a series of hierarchical multilevel regressions with trials nested within subjects. In a first step, we tested the effect of episodic retrieval of previous response information from the most recent occurrence of the same stimulus. Including response retrieval (coded as +.38 for same response trials vs. −.68 for different response trials) as the only level 1 predictor yields a large and highly significant retrieval effect, *β* = −60.73, *t*(25,542) = −39.58, *p* < .001. On average, participants responded 61 ms faster when the most recent occurrence of the word required the same response compared with trials where it required a different response.

Next, we added the interaction between the number of previous repetitions of color and word and response retrieval to the model. We obtained a significant product term, *β* = −23.72, *t*(25,542) = −7.24, *p* < .001, indicating that on average, the response retrieval effect was 24 ms larger after 10 prior repetitions compared with 1 prior repetition of color and word.^5^

Finally, we tested whether repeating the same S-R combination eventually leads to a stable association that influences responding independently of the most recent occurrence. We examined whether the response retrieval effect of a single episode is weakened by a series of preceding S-R episodes in which the word stimulus was paired with a different color. We included only probe trials in which the color-word combination presented during the last occurrence of the word repeated only once (57.7% of all trials). To increase statistical power and provide the most stringent test of our hypothesis, we contrasted trials in which the most recent occurrence of the word was preceded by only 1 earlier occurrence against trials in which it was preceded by 10 or 11 earlier occurrences of the word in a different color.

Including response retrieval as the only level 1 predictor produces a large and highly significant effect, *β* = −49.08, *t*(13,877) = −23.03, *p* < .001, indicating that participants responded 49 ms faster if the most recent occurrence of the word required the same response, compared with trials where the most recent occurrence of the word required a different response. We then added the interaction between the number of preceding mismatching episodes and response retrieval to the model. This interaction was not significant, *β* = 2.79, *t*(13,877) = 0.626, *p* = .531, indicating that the strength of the response retrieval effect was not affected by the length of the preceding series of presentations in which the word appeared in a different color.^6^ To further corroborate this result, we computed a *BF* comparing the model with the two-way interaction to the model without it. The *BF₀₁* indicated anecdotal evidence in favor of the simpler model without the interaction (*BF₀₁* > 1.96), suggesting that the data slightly favor the absence of a two-way interaction. In sum, these results suggest that the number of uninterrupted repetitions of a specific S-R combination did not have a lasting impact, as even a series of 10 or 11 episodes does not alter response binding and retrieval effects for an episode that is contrary to the series.

### Discussion

Experiment 2 featured longer sequences of color-word repetitions, yet produced the same pattern of results as Experiment 1: A substantial response retrieval effect that was amplified by the number of preceding matching episodes was obtained, but this repetition effect was eliminated by a single episode contrary to the series. Overall, Experiment 2 replicated the results of Experiment 1.

## General discussion

The primary objective of the present study was to empirically test the hypothesis that transient episodic bindings between stimulus and response features serve as the foundational elements for forming long-term S-R associations in memory (Frings et al., 2023). First, within the present study, we observed a substantial stimulus-response binding and retrieval effect: Participants responded faster if the most recent occurrence of the same stimulus required the same response, compared with trials where the most recent occurrence of the stimulus required a different response. Second, this response retrieval effect increased linearly with the number of prior identical S-R repetitions. Third, the modulating effect of an uninterrupted series of repetitions on binding and retrieval is fully eliminated after a single intervening episode that is contrary to the series.

### Theoretical implications

The fact that response retrieval effects increase with the number of prior identical repetitions of color and word is a novel and noteworthy finding. When interpreting this finding, it has to be taken into account, however, that the modulating effect of the number of repetitions is limited to modulating the binding and retrieval effects for an episode that is consistent with the repeated S-R combination, as there is no modulation of binding and retrieval effects for S-R combinations in which the word stimulus is paired with a different color. This lack of modulation for inconsistent S-R combinations rules out an explanation of the results in terms of long-term learning. Repeating a specific S-R combination apparently does not lead to establishing a long-lasting, nonepisodic representation that is independent of time or mismatching experiences. We found no convincing evidence that S-R repetitions accumulate to form an abstract, nonepisodic representation that operates independently from retrieval (e.g., an association or temporary proposition).

This raises the question of what drives the modulation of binding and retrieval effects for episodes that are consistent with the previous series of S-R episodes. In our view, larger S-R binding and retrieval effects after a series of homogeneous repetitions of the same S-R combination reflect more efficient retrieval processes. According to this explanation, the increase in the strength of S-R binding and retrieval effects results from a higher likelihood that the respective S-R combination is retrieved from memory if the previous occurrences of the word also required the same response. Specifically, episodic retrieval processes are characterized by some kind of noise or randomness. Although typically, the last occurrence of a stimulus is retrieved with the highest likelihood (the “law of recency”; Giesen et al., 2020; Schmidt, Giesen et al., 2020a, 2020b), this is not a deterministic process. Thus, in some situations, the last episode may be bypassed, or may not have been stored in memory, or might lose the race against other episodes that are temporally close, similar, or more salient. Increasing the number of identical recent episodes for a word, all paired with the same response, however, will increase the likelihood that one of these is found and retrieved, which will increase the estimate for the retrieval effect.

If episodic S-R bindings truly serve as the foundational building blocks for the formation of long-term S-R associations in memory, the accumulation of S-R episodes should have a lasting impact on behavior, even in the presence of a single intervening episode. However, since the effect of a preceding series of closely timed S-R repetitions modulates only retrieval effects for another matching S-R episode, and is eliminated by just one mismatching episode, it is unlikely that repetition facilitates the transition of episodic S-R bindings into long-term S-R associations within semantic memory (Logan, 1988; Logan & Etherton, 1994). Rather, the present results add to the evidence that binding and learning should be considered as two distinct processes (Colzato et al., 2006; Dames et al., 2022; Moeller & Frings, 2017), which operate independently from each other and have unique characteristics.

### Limitations and suggestions for future research

In Experiment 1, same response and different response trials were perfectly balanced, meaning they occurred with equal frequency, although the sequence length was relatively short (i.e., up to six repetitions). Although the design in Experiment 2 was not perfectly balanced (with more same response than different response trials), the results clearly show that the findings of Experiment 1 also hold for longer sequences (i.e., 10 or 11 repetitions). Still, the overrepresentation of long repetition sequences might have led participants to develop a strategy to expect repetitions rather than changes from the last color-word pairing, which might have led to an overestimation of the general retrieval effect (H1). Importantly, however, such expectancy-based strategies are independent of the length of the preceding series of identical repetitions, and thus does not bias the tests of the central hypotheses of our study (H2 and H3). Note that the imbalance in frequency between same and different response trials is difficult to avoid when testing longer sequences, as implementing a balanced design would not be feasible due to the excessive duration this would impose on the experiment. Most importantly, both factors (sequence length and response relation) and their interaction were considered simultaneously in the analysis, so that potential confounds were statistically controlled.

It is possible that repeated episodic S-R bindings contribute to the formation of abstract, nonepisodic representations, but that our current trial-by-trial test procedure may not be sensitive enough to detect such effects (Collins & Shanks, 2002). As there are only short-term repetitions of color and word in the current paradigm, implementing an awareness assessment would require on-task monitoring (i.e., multiple assessments after sequences). This, in turn, carries the risk of inducing strategies of propositional reasoning and hypothesis testing in participants, which would run counter to investigating pure effects of S-R repetitions; for this reason, we refrained from including measures of contingency awareness in our study. Thus, examining whether episodic bindings accumulate into integrative or propositional judgements lies beyond the scope of the present study and is incompatible with investigating pure effects of repetitions in the absence of higher-order reasoning processes.

Note, however, that we still observed measurable effects in the reaction time task, specifically, substantial response retrieval effects, which were further amplified by the number of prior repetitions of the same color-word pairings. It is thus unlikely that the paradigm is unsuitable for capturing these effects, as we were able to observe them even across a relatively short series of consecutive repetitions. Although this effect of the sequence length on episodic response retrieval was obtained robustly in our data in both experiments, it was completely eliminated after a single mismatching episode. If participants had formed nonepisodic “associative” representations, we would expect consistent behavioral facilitation beyond a single mismatching episode. Thus, we interpreted the lack of enduring behavioral effects for repeated S–R combinations as evidence against a contribution of episodic binding and retrieval to the formation of lasting, abstract S–R representations.

In our study, we investigated the effects of successive repetitions on binding and retrieval effects only for up to 10 successive repetitions. We did not investigate highly frequent repetitions (e.g., 1,000 times), as it is common in overlearning studies (e.g., Adams, 1982; Dickinson, 1985; Schmidt, De Houwer, et al., 2020a, 2020b), nor did we test the impact of repetitions across large timespans (e.g., Rothermund et al., 2025). Therefore, we cannot yet conclude that repetition never leads to the formation of abstract, nonepisodic representations. It also remains unclear whether increasing the length of a series of identical S-R repetitions will always continue to increase stimulus-response binding and retrieval effects, which is unlikely if the effect reflects more efficient retrieval as we suggested. Although these questions cannot finally be answered with the current data, the color-word repetition paradigm that we introduced in this study provides researchers with a promising method to investigate these questions in future studies. Specifically, testing the effects of repetition on retrieval effects for mismatching episodes provides a powerful tool to test the emergence of robust, long-term S-R representations.

## Data Availability

The experiments and data are publicly available on the Open Science Framework: https://osf.io/p45ye/?view_only=86367fc25b0c48f5bbb98b7baa8a9319

## Footnotes

1 Note that in the current paradigm, the target color always determines the response. Hence, it is possible that subsequent presentations of the word retrieve previous responses (i.e., left or right key press; S-R retrieval), previous targets (i.e., red or green; S-S retrieval), or both. Possible effects may therefore reflect S-S or S-R retrieval. However, for the present study, we are not specifically concerned with disentangling S-S and S-R retrieval, as our focus is on the overall influence of prior repetitions (for an experimental approach to disentangle S-S and S-R learning, see Schmidt et al., 2007, Experiment 4; for disentangling S-S and S-R retrieval, see Rudolph & Rothermund, 2025).

2 For instance, the last trial of the 20 sequences with a length of four was always followed by a trial in which the word was presented in a different color (otherwise it would not have been the last trial of the sequence), while the fourth trial of the 20 sequences with length of five and six was by definition always followed by a trial in which the word was presented in the same color.

3 The specific weights then depend on the frequency with which each condition appears in the data set:

$${w}_{1}=\frac{{f}_{2}}{\left({f}_{1}+{f}_{2}\right)}\text{ and} {w}_{2}=\frac{-{f}_{1}}{\left({f}_{1}+{f}_{2}\right)}$$

Equal trial frequencies in each condition will result in a contrast coding of *w*_*1*_* =* .5 and *w*_2_ = −.5. If the frequencies are unequal, however, this will result in unequal weights, so that the condition with the higher frequency receives a lower (absolute) weight (and vice versa). Thus, the resulting regression coefficient reflects the difference between the two conditions (in ms), whereby negative values for the regression coefficients indicate faster responses in the first condition (e.g., same response trials).

4 To control for effects of mere response repetitions, we included additional predictors coding the response match/mismatch, sequence length, and their interaction for the last occurrence of the *other* word that did not correspond to the distractor in the current trial into the analysis. This analysis revealed a small but significant effect for the response relation factor, indicating pure response priming, *β* = −8.19, *t*(103,734) = 11.84, *p* < .001, but no interaction with sequence length, *β* = −0.88, *t*(103,734) = −1.50, *p* = 0.133. Importantly, the effects of the distractor word in the current trial were unaffected by including these additional predictors. Notably, the response relation factor showed an effect three times larger for the matching word, and its robust interaction with the corresponding sequence length indicates that the effects are due to episodic response retrieval rather than mere response priming.

5 We also included additional predictors coding response match/mismatch, sequence length, and their interaction for the last occurrence of the *other* word into the analysis to control for effects of mere response repetitions. This analysis revealed a significant effect for the response relation factor, indicating pure response priming, *β* = −38.49, *t*(25,264) = −26.09, *p* < .001, but no interaction with sequence length, *β* = −6.76, *t*(25,264) = -1.03, *p* = 0.301. Similar to Experiment 1, the effects of the distractor word in the current trial were unaffected by including these additional predictors. Although Experiment 2 involved up to 10 repetitions of the word in the same color, the response relation factor showed an effect twice as large for the matching word, and its robust interaction with the corresponding sequence length indicates that the effects are due to episodic response retrieval rather than mere response priming.

6 We checked for possible differences between participants in Experiment 2, as some were psychology students while others were recruited via Prolific. No significant differences were found for the effects of interest between the groups (all t ≥ 0.645, all *p* ≥ 0.519).

## Code availability

The R analysis scripts are publicly available on the Open Science Framework (https://osf.io/p45ye/?view_only=86367fc25b0c48f5bbb98b7baa8a9319). More information is available from the lead author on request.
